# NO_2_– Mediates the Heart Protection of Remote Ischemic Preconditioning

**Published:** 2016-02-01

**Authors:** M. S. A. Mohamed

**Affiliations:** Thoracic Transplantation Department, University Clinic Essen, Germany

Ischemic preconditioning is a technique where prior application of repeated short cycles of ischemia and reperfusion would be able to attenuate the severity of the subsequent ischemic reperfusion injury (IRI). Remote ischemic preconditioning (RIPC) describes the ability of the technique to function through distance. For example, the application of short, repetitive ischemia-reperfusion cycles of the limb would protect distant organs like heart, kidney, brain, and liver during subsequent IRI. Both phenomena indicate the involvement of local, paracrine, as well as remote circulating mediators [1].

During limb ischemia, the diminished flow and shearing stress would be associated with cell membrane depolarization and inhibition of the inward driving K^+^ channels. The inhibition of KATP channels would lead to the activation of T type Ca^2+^ channels and increased Ca^2+^ influx into endothelial cells. Increased intracellular Ca^2+^ activates Ca^2+^-dependent endothelial NO synthase (eNOS) [1, 2]. Simultaneously, hypoxia and ischemia would result in an increased production of reactive oxygen species (ROS). Hypoxia inhibits oxidative phosphorylation and results in decreased ATP production. That activates xanthine oxidase, leading to increased ROS production. The inhibition of KATP channels, and the persistence of cell membrane depolarization would result in NADPH oxidase (NOX2) activation, leading to more increase in ROS production [1, 3]. Increased production of both NO and ROS would be associated with NO oxidation to produce nitrite (NO_2_^–^). 

Several studies documented the important role of NO in mediating the protective effect of IPC and RIPC. While the locally produced NO can exert its action in case of IPC, it cannot be accused for RIPC protective effect because of its short blood half-life (≤ 2 ms) [4]. However, it was observed that NO inhalation in human provides protection against IRIs, while being associated with a significant increase in the circulating levels of nitrite. In addition, NO_2_^–^ showed the ability to protect against IRI, to exert cytoprotective effects, and to decrease the infarction size similar to NO [5-12]. Moreover, it has recently been confirmed that the application of brachial artery RIPC results in the activation of eNOS and increased plasma NO_2_^–^ levels [13].

In the heart, NO_2_^–^ would be reduced to NO and N_2_O_3_ by myoglobin [14, 15]. NO and S-nitrosothiols formed from nitrite would inhibit complex I of the respiratory chain during reperfusion. This would attenuate the increased production of ROS in response to IRI, and would indirectly affect the functionality of complex II [16, 17]. Being at cross-talking with mitochondrial KATP channels, modification of the functional activity of complex II would influence the activity of mitochondrial KATP channels [18], this might contribute to an improved activity of these channels in response to RIPC, which would inhibit the opening of mitochondrial permeability transition pores and the subsequent release of cytochrome c during reperfusion [17, 19].

An important mechanism in the development of the IRI is the increased production of inflammatory cytokines, which would be responsible for the recruitment of inflammatory cells and initiation of adverse inflammatory reactions [20]. In addition to the significant increase in ROS production, IRI activates toll-like receptors [TLRs]. Both result in priming of the heart inflammasomes [21]. 

During ischemia and hypoxia, as well as cold preservation of the heart graft, the associated inhibition of Na^+^-K^+^ ATPase and other K^+^ channels would result in decreased intracellular K^+^ levels. Even with the administration of high extracellular K^+^ concentrations (during cardioplegia), this would lead to the closure of K^+^ channels [3]. The end-result would be the drop in intracellular K^+^ levels, which activates the primed inflammasomes [22]. 

**Figure 1:**
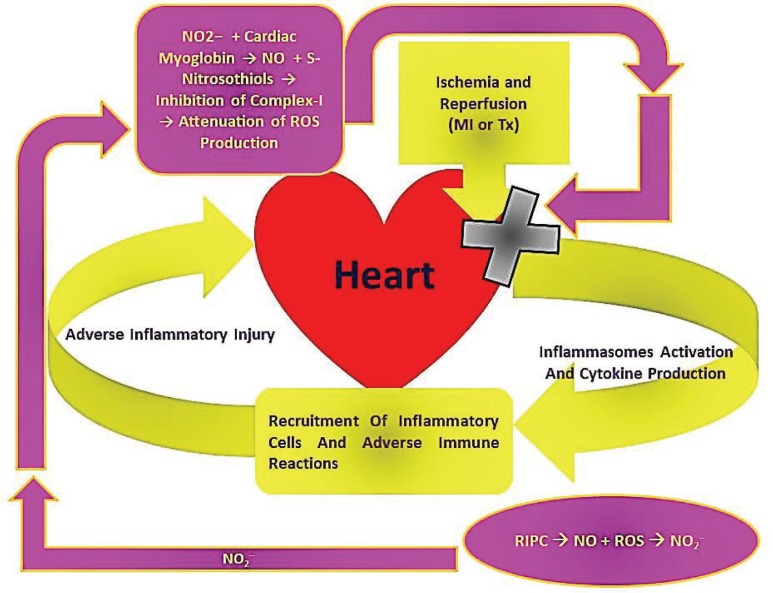
Diagrammatic representation of the mechanism, through which NO_2_^– ^generated in response to RIPC would be involved in the attenuation of inflammasomes activation and cytokine production within the heart in response to IRI

Activated inflammasomes activate caspase-1, which activates proIL1β and proIL18, which are able to induce IL6. With the important role of inflammasomes and TLRs in the establishment of the inflammatory reactions of the IRI, the above-described role of NO and NO_2_^–^ to attenuate ROS production and to improve the activity of KATP channels would interfere with inflammasomes priming and activation in response to IRI. Accordingly, this would contribute to decreased production of inflammatory cytokines, which would ultimately attenuate the immune cell infiltration and the adverse immune reactions generated in response to the IRI (Fig 1).

This mechanism of action highlights the importance of inactivation of inflammasomes, through RIPC, for the attenuation of the hazards of IRI. Although it was reported, to the contrary, by some studies that the deletion of NLRP3, which is the most studied inflammasome component, abates the protective effects of IPC due to the inhibition of IL6 production and lack of its signaling [23].

It seems that various inflammatory cytokines are involved in the stimulation of the adverse inflammatory reactions in response to IRI, as well as, in protective feedback signaling against subsequent IRI. Accordingly, the above-mentioned scenario should be confirmed as a whole by experimental studies to identify whether blocking the release of IL1β and IL18, with the subsequent lack of IL6 induction, would increase or decrease heart protection in response to RIPC.

Nevertheless, the augmentation of the above-presented scenario at different levels (*e.g.*, through NO inhalation, NO_2_^–^ administration, or the use of KATP channel agonists) prior to heart transplantation, and or other forms of cardiac IRI, was found to provide a significant degree of protection, with associated better clinical outcomes [24]. 

Further studies should be conducted to confirm this mechanism, and whether it could also be considered for other organs such as lung, kidney and liver. 
